# Changing contribution of smoking to the sex differences in life expectancy in Europe, 1950–2014

**DOI:** 10.1007/s10654-020-00602-x

**Published:** 2020-01-22

**Authors:** Fanny Janssen

**Affiliations:** 1 Faculty of Spatial Sciences, Population Research Centre, University of Groningen, P.O. Box 800, 9700 AV Groningen, The Netherlands; 2 Netherlands Interdisciplinary Demographic Institute - KNAW / University of Groningen, P.O. Box 11650, 2502 AR The Hague, The Netherlands

**Keywords:** Smoking, Smoking-attributable mortality, Life expectancy, Sex differences, Europe

## Abstract

This article provides a detailed and overarching illustration of the contribution of smoking to sex differences in life expectancy at birth (e0) in Europe, focusing on changes over time and differences between both European countries and European regions. For this purpose, the sex difference in e0 for 31 European countries over the 1950–2014 period was decomposed into a smoking- and a non-smoking-related part, using all-cause mortality data and indirectly estimated smoking-attributable mortality rates by age and sex, and a formal decomposition analysis. It was found that smoking-attributable mortality contributed, on average, 3 years (43.5%) to the 7-year life expectancy difference between women and men in 2014. This contribution, was largest in 1995, at 5.2 out of 9.0 years, and subsequently declined in parallel with the average sex difference in life expectancy. The average contribution of smoking-attributable mortality was especially large in North-Western Europe around 1975; in Southern Europe around 1985; and in Eastern Europe around 1990–1995, when smoking-attributable mortality reached maximum levels among men, but was still low among women. The observed parallel decline from 1995 onwards in the sex differences in e0 and the absolute contribution of smoking to this sex difference suggests that this recent decline in the sex difference in e0 can be almost fully explained by historical changes in sex differences in smoking, and, consequently, smoking-attributable mortality. In line with the progression of the smoking epidemic, the sex differences in life expectancy in Europe are expected to further decline in the future.

**Electronic supplementary material:**

The online version of this article (10.1007/s10654-020-00602-x) contains supplementary material, which is available to authorized users.

## Introduction

Sex differences in life expectancy at birth are large and seem persistent. For Europe, in 2015, the life expectancy of a European man was, on average, 6.6 years lower than the life expectancy of a European woman [1]. To understand how persistent these sex differences are and how they are likely to develop in the future, a detailed investigation of changes over time in the contribution of smoking, one of its main determinants, is warranted.

The smoking behaviours of men and women have differed substantially in Europe, with men taking up smoking much earlier and more extensively than women [2]. These patterns have resulted in distinct sex differences in smoking-attributable mortality emerging around three decades later, with smoking-attributable mortality being higher among men than women, although this gap has been changing over time [3, 4].

Previous studies that quantified the contribution of smoking to sex differences in life expectancy [5–7] found that smoking indeed played an important role. Waldron estimated a contribution of smoking to sex differences in adult mortality of about 50 percent, using survey data mainly for the United States from the late 1950s–1980 [5]. McCartney et al. estimated the contribution of smoking-related causes of death to the gender gap in mortality to be generally around 40% to 60% for 30 European countries around 2005 [6]. Luy and Wegner-Siegmundt analysed the average impact of smoking-attributable mortality on sex differences in life expectancy over the period 1955/59–2005/09 in 53 industrialised countries, which, in Europe, varied substantially from 8.9% in Iceland to 62.5% in the Netherlands [7]. They also showed that, in most industrialized countries, trends over time in the sex differences in life expectancy can be attributed to smoking.

This short report will provide a detailed and overarching illustration of the contribution of smoking to sex differences in life expectancy in Europe, based on the latest available data (1950–2014). In contrast to previous studies, I will focus on examining changes over time in the role of smoking in the sex gap in life expectancy, and the differences therein between both European countries and European regions.

## Methods

Sex differences in life expectancy at birth (e0) for the national populations of 31 European countries, both individually and combined, for the years 1950 up until 2014 are estimated by applying standard demographic life table techniques to age- and sex-specific data on all-cause death numbers and population exposures, obtained from the Human Mortality Database (www.mortality.org). The sex differences in e0 are subsequently decomposed into the differences due to smoking-attributable mortality versus non-smoking-attributable mortality using the decomposition technique by Andreev et al. [8] Smoking-attributable mortality is estimated by means of the validated adjusted indirect Peto et al. method [9, 10], which uses national lung cancer mortality data [which we obtained from WHO (www.who.int/healthinfo/statistics/mortality_rawdata)] as a proxy for lifetime smoking prevalence, and takes into account other smoking-attributable deaths using additional epidemiological information. See as well the Supplementary Materials and Methods.

## Results

**Fig. 1:**
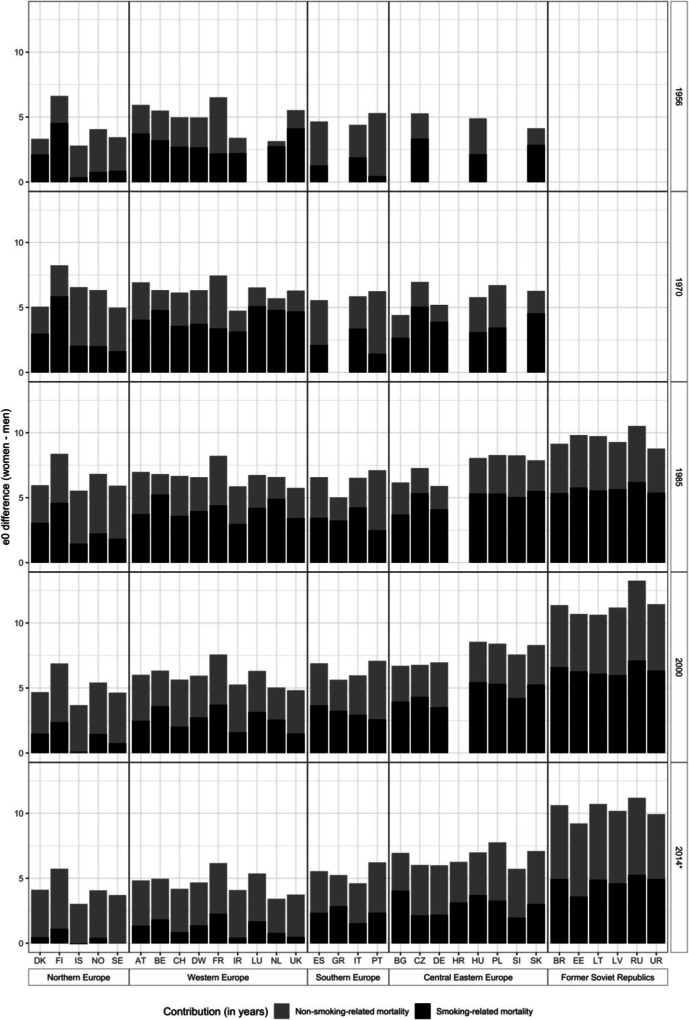
The contribution of smoking to the sex difference in life expectancy at birth (e0), 31 European countries, 1956–2014*, selected years. *Latest available year for Bulgaria (2010), Greece (2013), Ukraine (2012) and Russia (2013). See notes Table 1 for full country names

In 2014, the sex difference in e0 was, on average, 7.0 years across the 31 European countries studied, but ranged from 11.2 years in Russia to 3.0 years in Iceland (Fig. 1 (bottom), Table S1). Smoking-attributable mortality contributed, on average, 3.0 years (43.5%) to the sex difference in e0. The absolute contribution of smoking-attributable mortality was highest in Russia (5.3 years) and lowest in Sweden (zero) and Iceland (− 0.1). The relative contribution of smoking-attributable mortality was 50% or higher in Greece, Bulgaria, Croatia, Hungary, and Ukraine. On average, the contribution of smoking-attributable mortality to the sex difference in e0 was 4.6 years (47.5%) in Eastern Europe, 2.0 years (39%) in Southern Europe, 1.3 years (28%) in Western Europe, and 0.4 years (10%) in Northern Europe (Table 1).

**Table 1:** Sex difference in life expectancy at birth (e0), and the absolute and relative contribution of smoking-attributable mortality, European regions*, 1956–2014**, selected years

	1956	1960	1965	1970	1975	1981	1985	1990	1995	2000	2005	2010	2014**
Sex difference in e0													
Europe	*5.13*	*5.52*	5.95	*6.24*	*6.59*	*8.16*	7.82	7.99	9.04	8.66	8.57	7.46	6.97
Non-Eastern Europe	*5.14*	*5.52*	*6.02*	*6.30*	*6.60*	6.70	6.66	6.64	6.51	6.06	5.61	5.18	4.82
Northern Europe	4.12	4.44	5.21	5.91	6.48	6.68	6.60	6.27	5.83	5.29	5.01	4.66	4.29
Western Europe	*5.48*	*5.80*	*6.25*	6.54	6.74	6.84	6.76	6.64	6.45	5.99	5.47	5.08	4.72
Southern Europe	*4.58*	*5.09*	*5.61*	*5.79*	*6.28*	6.41	6.46	6.68	6.77	6.35	5.99	5.46	5.11
Eastern Europe	*4.97*	*5.42*	*5.48*	*6.04*	*6.51*	*9.88*	9.24	9.62	11.62	11.38	11.64	10.2	9.69
CEE (not former USSR)	*4.97*	*5.42*	*5.48*	*6.04*	*6.51*	*7.22*	7.48	8.23	8.25	7.81	7.69	7.38	6.98
Former Soviet Republics	NA	NA	NA	NA	NA	10.99	10.01	10.2	12.87	12.71	13.08	11.38	10.86
Absolute contribution of smoking-attributable mortality (years) to the sex difference in e0													
Europe	*2.56*	*2.86*	*3.32*	*3.66*	*3.95*	*4.76*	4.75	4.73	5.23	4.76	4.46	3.54	3.03
Non-Eastern Europe	*2.55*	*2.93*	*3.36*	*3.63*	*3.89*	3.92	3.86	3.56	3.22	2.73	2.28	1.81	1.48
Northern Europe	1.88	2.28	2.63	2.89	2.93	3.00	2.75	2.37	1.85	1.40	1.04	0.72	0.43
Western Europe	*3.04*	*3.40*	*3.84*	4.05	4.24	4.17	4.03	3.63	3.20	2.64	2.15	1.65	1.32
Southern Europe	*1.54*	*1.91*	*2.35*	*2.77*	*3.29*	3.62	3.76	3.68	3.55	3.16	2.78	2.32	1.99
Eastern Europe	*2.73*	*2.45*	*3.12*	*3.74*	*4.19*	*5.62*	5.64	5.86	6.80	6.38	6.22	5.16	4.60
CEE (not former USSR)	*2.73*	*2.45*	*3.12*	*3.74*	*4.19*	*4.71*	4.93	5.19	5.15	4.69	4.29	3.62	3.00
Former Soviet Republics	NA	NA	NA	NA	NA	5.96	5.93	6.14	7.32	6.89	6.76	5.65	5.14
Relative contribution of smoking-attributable mortality (%) to the sex difference in e0													
Europe	*49.92*	*51.85*	*55.82*	*58.56*	*60.02*	*58.33*	60.68	59.24	57.91	55.02	52.1	47.47	43.48
Non-Eastern Europe	*49.5*	*53.07*	*55.76*	*57.65*	*58.88*	58.52	57.86	53.61	49.39	44.99	40.63	34.97	30.6
Northern Europe	45.70	51.27	50.61	48.95	45.27	44.89	41.75	37.81	31.64	26.47	20.87	15.54	10.08
Western Europe	*55.46*	*58.69*	*61.50*	62.00	62.88	60.95	59.59	54.62	49.6	44.16	39.23	32.57	27.86
Southern Europe	*33.58*	*37.49*	*41.88*	*47.76*	*52.39*	56.43	58.09	55.17	52.48	49.86	46.35	42.47	38.93
Eastern Europe	*54.84*	*45.13*	*56.93*	*61.96*	*64.48*	*56.91*	61.02	60.93	58.54	56.09	53.44	50.57	47.45
CEE (not former USSR)	*54.84*	*45.13*	*56.93*	*61.96*	*64.48*	*65.27*	65.93	63.14	62.48	60.04	55.7	49.11	42.99
Former Soviet Republics	NA	NA	NA	NA	NA	54.26	59.31	60.25	56.89	54.2	51.69	49.64	47.37

^*^Croatia (HR) was excluded for comparability purposes. This led to differences only in the second decimal. The number of countries that could be included differs per year. 1956 and 1960: 20 countries (Germany West instead of Germany as a whole); 1965: 21 countries (Germany West instead of Germany as a whole); 1970 and 1975: 23 countries; 1981: 29 countries; 1985 onwards: 30 countries. In italics are the numbers that can not be compared over time because of this.

^**^Latest available year for Bulgaria (2010), Greece (2013), Ukraine (2012), and Russia (2013)

Europe: all countries included in the analysis*

Eastern Europe: all Central and Eastern European countries included in the analysis*

Non-Eastern Europe: all Northern, Western and Southern European countries included in the analysis

Northern Europe: Denmark (DK), Finland (FI), Iceland (IS), Norway (NO), Sweden (SE)

Western Europe: Austria (AT), Belgium (BE), Germany West (DW), France (FR), Ireland (IR), Luxembourg (LU), Netherlands (NL), Switzerland (CH), United Kingdom (UK)

Southern Europe: Greece (GR), Italy (IT), Portugal (PT), Spain (ES)

Central Eastern Europe (CEE) (without former USSR): Bulgaria (BG), Czech Republic (CZ), Germany East (DE), Hungary (HU), Poland (PL), Slovakia (SK), Slovenia (SI)

Former Soviet Republics: Belarus (BR), Estonia (EE), Latvia (LV), Lithuania LT), Ukraine (UR), Russia (RU)

However, neither the sex differences in e0 nor the contribution of smoking-attributable mortality have been stable over time.

In 1985, the sex difference in e0 was higher in all the 30 individual countries, except in Greece, East Germany, Bulgaria, Belarus, Latvia, Lithuania, Russia, and Ukraine (Fig. 1). The contribution of smoking-attributable mortality was higher in 1985 in all countries, both in absolute (not Bulgaria) and relative terms (not Portugal). In 1956, however, the sex difference in e0 was smaller than it was in 2014 in the majority of countries; whereas the contribution of smoking-attributable mortality was larger, except in France, Portugal, Spain, and Hungary.

The average sex difference in e0 was 7.8 years in 1985 and was highest in 1995 at 9.0 years (Table 1). The average relative contribution of smoking-attributable mortality to the sex differences in life expectancy was almost 60% around 1985, after which it declined. The absolute contribution of smoking-attributable mortality was, on average, highest around 1995 at 5.2 years, after which it declined in parallel with the declining trend in the sex difference in e0.

**Fig. 2:**
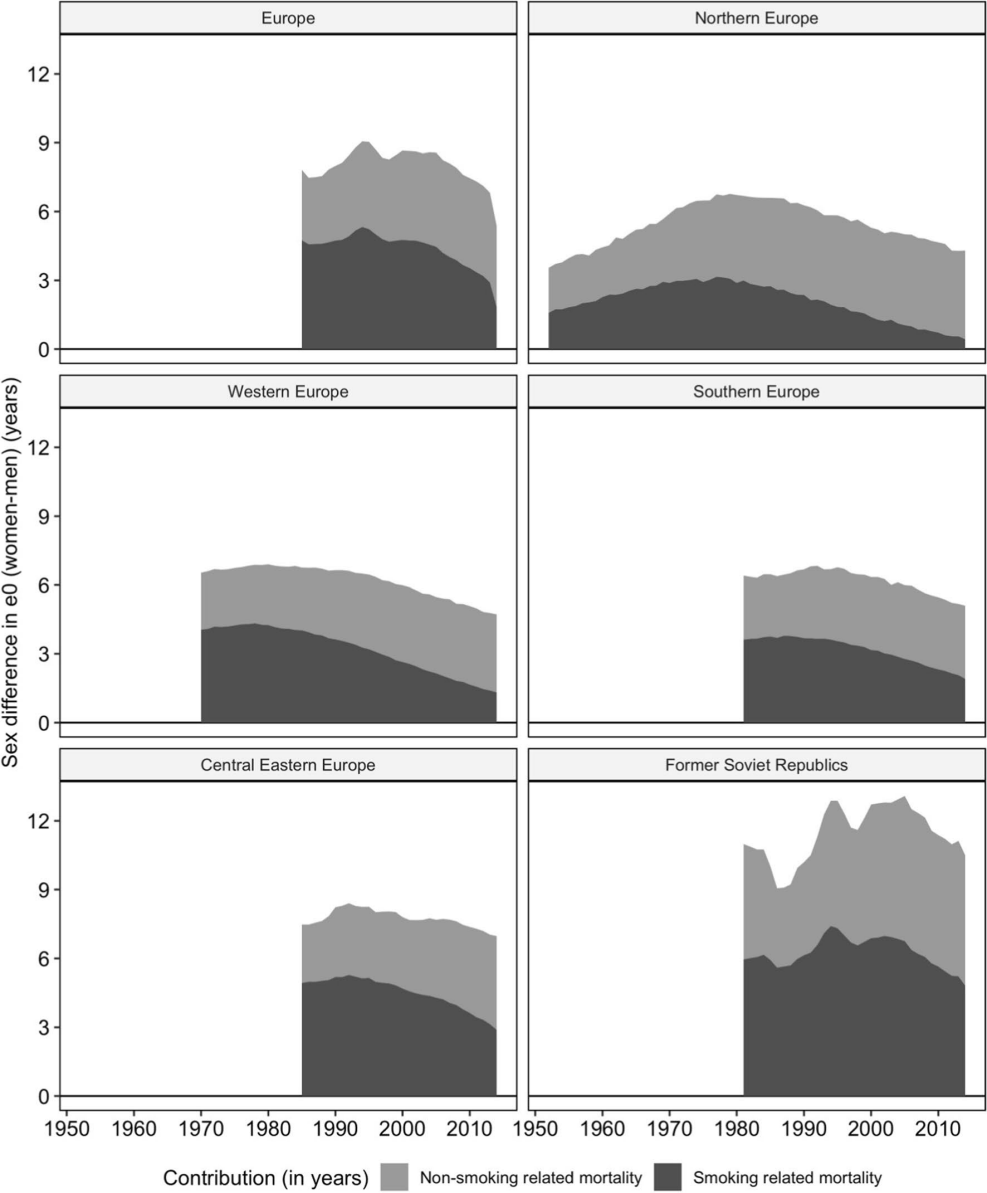
Changes over time in the absolute contribution of smoking-attributable mortality to the sex difference in life expectancy at birth (e0), European regions (See the notes regarding Table 1), 1950–2014 (Only the years are included for which the contributions can be compared over time)

The maximum average contribution of smoking-attributable mortality varied across Europe and was observed, on average, in Western Europe in 1975 (4.2 years), in Northern Europe in 1981 (3.0 years), in Southern Europe in 1985 (3.8 years), in Central Eastern Europe in 1990 (5.2 years), and in the former Soviet Republics in 1995 (7.3 years) (Fig. 2). The contribution of non-smoking-related mortality has remained stable at around 3.9 years since around 1995 (Fig. 2). In Northern Europe, such a stable trend already occurred from 1985 onwards; whereas in Eastern Europe, a stable trend is not yet clearly visible. In the former Soviet Republics, the contribution of non-smoking-related mortality was high throughout the study period (5.7 years in 2014) (Table S1); whereas in Southern and Western Europe, its contribution has been around 3.2–3.4 years since 1995. Figure S1 illustrates important country differences.

## Discussion

The average contribution of smoking-attributable mortality to the sex differences in life expectancy across 30 European countries declined from 5.2 out of 9.0 years (58%) in 1995 to 3.0 out of 7.0 years (43.5%) in 2014. This declining contribution closely resembles the declining sex differences in smoking-attributable mortality resulting from differential changes in smoking behaviour about 30–40 years earlier, when the health risks of smoking had become broadly known [3, 4]. Around 1955, men in high-income countries reached maximum smoking prevalence levels (50–80%), after which widespread smoking cessation resulted in strong declines. For women, however, who took up smoking later than men, smoking prevalence levels were still increasing slightly, reaching 35–45% around 1965 [3, 4].

The observed substantial differences between regions and countries in the year in which the contribution of smoking-attributable mortality was at its maximum, are consequently predominantly due to differences in the timing of the peak of smoking-attributable mortality among men, which occurred earlier in North-Western Europe compared to Southern and Eastern European countries [10].

The observed parallel decline from 1995 onwards in the sex differences in life expectancy and the absolute contribution of smoking-attributable mortality to this difference suggests that this recent decline in the sex differences in e0 can almost fully be explained by the abovementioned changing sex differences in smoking-attributable mortality. The increase in the sex differences in e0 before 1995 also seems to be largely driven by the increase in smoking-attributable mortality among men. In this period, however, increases in the contribution of non-smoking-related mortality also occurred in the majority of countries. This indicates the presence of an additional effect of other non-biological factors on sex differences in life expectancy, such as other behavioural risk factors (e.g., alcohol use, physical activity, diet, accidents, violence, suicide), as well as social, cultural, and economic factors [7]. Given that social and economic inequality between men and women has, in general, not increased in Europe since 1950, the observed increase in the contribution of non-smoking-related mortality most likely points to increased sex differences in (some of) the abovementioned other behavioural risk factors.

The country differences in the trend since 1995 in the contribution of non-smoking-attributable mortality likely point to a different balance of trends in sex differences in the differential risk behaviours other than smoking. For example, European men generally experienced slightly stronger increases in obesity-attributable mortality than European women, but more so in Eastern than in Western Europe [11]. Whereas in the United Kingdom, the Netherlands, Belgium, and most Former Soviet Republics (up to 2000), men have experienced stronger increases in alcohol-attributable mortality than women, in Hungary, declines in alcohol-attributable mortality have been greater among men than among women since 1995 (own unpublished results).

The higher absolute contribution of non-smoking-related mortality observed in Former Soviet Republics and Finland may be related to the much higher and riskier alcohol consumption levels among men than among women, and to the subsequent sex differences in alcohol-attributable mortality [12]. When the contribution of non-smoking-related mortality approaches 2.0 years, this could indicate that only the biological component of the sex difference in life expectancy remains, which ranges from 0.5 to 2.0 years [7].

In line with the progression of the smoking epidemic [3, 4], the sex differences in life expectancy are expected to (further) decline in the coming decades as a consequence of decreasing smoking-attributable mortality among men, coupled with generally increasing smoking-attributable mortality among women [10]. This decline in the sex difference in life expectancy is expected to be stronger among Southern European and Eastern European countries, as the peak in smoking-attributable mortality among women will likely be reached at a later point in time in these countries than in North-Western European countries [10]. As a consequence, the differences between countries in the sex gap in life expectancy will likely also diminish.

Thus, the sex difference in life expectancy will be driven less and less by smoking, and more and more by other behavioural risk factors. Additional research is warranted to assess the likely future development of the sex difference in life expectancy that is not due to smoking. Given that these risk factors have a smaller impact on life expectancy than smoking, and that trends in sex differences in these factors might balance each other out, an effect that is as large as that of smoking on the evolution of the sex difference in e0 over time is not expected.

## What is already known

Sex differences in life expectancy at birth (e0) in Europe are large, and these differences are affected to a large extent by smoking. What this information tells us about the persistence of sex differences in e0 and how they will develop in the future is unknown.

## What this study adds

- The detailed examination of changes over time (1950 up to 2014) in the contribution of smoking-attributable mortality to the sex differences in life expectancy revealed that the average contribution of smoking-attributable mortality to the sex differences in life expectancy across 30 European countries was greatest in 1995, at 5.2 out of 9.0 years (58%), after which it declined to 3.0 out of 7.0 years (43.5%) in 2014.
- The decline in the average sex difference in life expectancy since 1995 across 30 European countries (from 9 to 7 years) can almost fully be explained by the declining sex differences in smoking-attributable mortality, which mainly result from sharp declines in smoking prevalence among men about 30–40 years earlier.
- The average contribution of smoking-attributable mortality was especially large among countries and regions during periods when smoking-attributable mortality rates were close to its peak among men, but were still low among women
- The sex differences in life expectancy are expected to (further) decline in the coming decades, but not to disappear given the important contribution of non-smoking-attributable mortality to the sex difference.

## Electronic supplementary material

Below is the link to the electronic supplementary material.

Supplementary file1 (DOCX 29 kb)

Supplementary file2 (DOCX 678 kb)

## Data Availability

The data that were used as input for the tables and figures will be made available at Open Science Framework. The R code can be requested from the author.
