# Supplementary material for: Changing contribution of smoking to the sex differences in life expectancy in Europe, 1950–2014
Source: Eur J Epidemiol. 2020 Jan 22;35(9):835–41. doi: 10.1007/s10654-020-00602-x (PMC7524860; doi:10.1007/s10654-020-00602-x)
Supplement: Supplementary file 1 — Supplementary file1 (DOCX 29 kb) [file 10654_2020_602_MOESM1_ESM.docx]

**Supplementary information – Materials and Methods**

**Setting / Data availability**

I included 31 European countries for which data on all-cause mortality and exposure are available from the Human Mortality Database (HMD)^1^ to ensure that the data on the sex differences in life expectancy are of high quality. For these countries, lung cancer mortality death numbers by sex and five-year age groups (0-4, … , 75-79, 80+) were also available from the WHO Mortality Database^2^. The national age- and sex-specific lung cancer mortality rates necessary for estimating smoking-attributable mortality (see further details below) were obtained by dividing the lung cancer death numbers from WHO by the exposure data from the HMD. For some years in some countries for which WHO data were missing or were not detailed enough, additional information on lung cancer mortality was obtained or additional calculations were applied. See Table 1 in this document for the final countries included and the final data availability. Based on the data availability I selected the years 1950 up until 2014 for the analyses. For those countries for which no data for 2014 was available yet, I selected instead the latest available year: Bulgaria 2010, Greece 2013, Ukraine 2012, Russia 2013. For Germany, I distinguished Eastern and Western Germany. In the comparison over time, I excluded Croatia for which data were available from 2002 onwards only. This hardly affected the average sex difference in life expectancy and the average contribution of smoking.

**Calculation of the sex differences in life expectancy**

By applying standard life table techniques^3^ to all-cause death numbers and population exposures by single year of age and sex obtained from the HMD, I estimated the sex differences in life expectancy at birth (e0) for the 31 European countries for the years 1956 up until 2014 (or, if the values for 2014 were not yet available, the values for the latest year available). I chose ages 100+ for the final open-ended age group in the life table calculations. In addition, weighted averages of the sex difference in e0 for all studied European countries combined and by European region were calculated, based on similar life table calculations applied to the sum of the death numbers and the sum of the population exposures over the individual countries.

**Estimation smoking-attributable mortality**

I estimated smoking-attributable mortality by country, year, sex, and age (35+) by applying a simplified indirect Peto-Lopez method.^4,5^ The method uses national lung cancer mortality rates as a proxy for lifetime smoking prevalence, making use of the fact that almost all lung cancer mortality is due to smoking. Subsequently, population-attributable fraction calculations are applied to this estimated smoking prevalence, while also information on the relative risks (RRs) of dying from smoking is used. By doing so, the method also takes into account the effects of smoking on causes of death, besides lung cancer mortality.

In the first step, the lifetime smoking prevalence (𝑝) by five-year age groups was estimated by comparing the observed national age- and sex-specific lung cancer mortality rates with the age- and sex-specific lung cancer rates of smokers and never smokers (smoothed) of the ACS CPS-II study.^4^ Lifetime smoking prevalence by single year of age was obtained by means of Loess smoothing (span = 0.75; degree = 2), with age 85 used as the central age for the age group 80+. From age 86 onwards I applied the smoothed lifetime smoking prevalence for age 85.

In the second step, I estimated the smoking-attributable mortality fractions (SAMF) by single year of age for all causes of death combined instead of by cause of death, as in the original Peto-Lopez method. The SAMF was calculated using the formula of the population attributable fraction: SAMF_x,s_ = 𝑝_x,s_ (RR_x,s_−1)/(𝑝_x,s_ (RR_x,s_−1)+1), where 𝑝_x,s_ reflects the obtained sex-specific estimates of the lifetime smoking prevalence by single year of age, and RR_x,s_ reflects the relative risks of dying from smoking by single year of age and sex. RRs by five-year age groups (35-39, 40-44, …, 80-84, 85+) and sex were obtained by dividing the all-cause mortality rates among CPS-II current smokers by the all-cause mortality rates among CPS-II never smokers.^6^ To control for the exposure of smokers to other risk factors, the excess risk was reduced by 30%.^7^ RRs by single year of age were obtained by applying a second-degree polynomial to the data up to age 89 for men and up to age 86 for women, with age 86 used as the central age for the age group 85+. For ages 90-100 among men and ages 87-100 among women, the RRs were kept constant to the smoothed value for age 89 for men and for age 86 for women.

Smoking-attributable death numbers and non-smoking-attributable death numbers by age, sex, country, and year are subsequently obtained by multiplying the respective SAMF by the respective all-cause deaths. By dividing them with the respective exposure, smoking-attributable mortality rates and non-smoking-attributable mortality rates by age, sex and year were obtained for the different individual countries. By summing the smoking-attributable death numbers and non-smoking-attributable death numbers over the individual countries for the different regions, and subsequently dividing them by the respective exposure, weighted averages of smoking-attributable mortality rates and non-smoking attributable mortality rates were obtained for the different regions and all countries combined.

**Decomposition technique**

To estimate the contribution of smoking to the sex differences in e0, the technique for decomposing differences in life expectancy, as described by Andreev et al. 2002,^8^ is applied to the all-cause, smoking-related and non-smoking-related mortality data by single year of age (0-100+), for the individual countries and the region totals.

That is, the sex differences in e0 are decomposed into the part that is due to smoking-attributable mortality and the part that is due to non-smoking-attributable mortality. The decomposition technique, described by Andreev et al. 2002,^8^ is, in essence, similar to the decomposition techniques developed independently by Andreev 1982,^9^ Arriaga 1984,^10^ and Pressat 1985.^11^ Unlike the often-applied Arriaga 1984 technique, the method described by Andreev et al. 2002 takes into account that different outcomes can result from using a different order of the populations (e.g., men – women versus women – men) by applying the method to the two different orders and averaging the results. ^8^

Applying the decomposition technique, as described by Andreev et al. 2002, to the objective of estimating the contribution of smoking to the sex differences in e0 involved the following steps for each country and year: (i) decomposing the sex differences in life expectancy into the contribution of each single year of age; (ii) assessing the relative contribution of smoking to the total mortality differences in each age by dividing the sex differences in smoking-attributable mortality rates by the sex differences in all-cause mortality rates; (iii) decomposing the age-specific contributions into the contribution of smoking-attributable mortality and the contribution of non-smoking-attributable mortality by multiplying the estimates obtained in step *i* with the estimates obtained in step *ii*; and (iv) summing up the age-specific contributions of smoking and non-smoking attributable mortality over all ages.

Among the advantages of using a decomposition technique rather than using life table techniques applied to non-smoking-related mortality are that, when applying decomposition, no hypothetical assumption about the complete removal of smoking-related mortality needs to be made; and that the contribution of smoking-related mortality and non-smoking-related mortality can be assessed simultaneously.

**References**

1. Human Mortality Database. University of California, Berkeley (USA), and Max Planck Institute for Demographic Research (Germany). Available at: www.mortality.org or www.humanmortality.de (data downloaded on 29-09-2018).
2. WHO Mortality Database. Health statistics and health information systems. available at: www.who.int/healthinfo/statistics/mortality_rawdata/en/ (update April 11, 2018).
3. Preston SH, Heuveline P, Guillot M. *Demography*. Malden, MA: Blackwell; 2001.
4. Peto R, Boreham J, Lopez AD, Thun M, Heath C. Mortality from tobacco in developed countries: indirect estimation from national vital statistics. *Lancet* 1992; **339**:1268­78.
5. Janssen F, Van Wissen, Kunst AE. Including the smoking epidemic in internationally coherent mortality projections. *Demography* 2013; **50**:1341­62.
6. Thun MJ, Day-Lally C, Myers DG, Calle EE, Flanders WD, Zhu BP. Trends in tobacco smoking and mortality from cigarette use in Cancer Prevention Studies I (1959 through 1965) and II (1982 through 1988). In: Burns DM, Garfinkel L, Samet JM, eds. *Changes in Cigarette-Related Disease Risks and Their Implications for Prevention and Control.* Bethesda, Md: National Cancer Institute; 1997; Smoking and Tobacco Control Monograph 8: 305-382.
7. Ezzati M, Lopez AD. Estimates of global mortality attributable to smoking in 2000. *Lancet* 2003; **362**:847-852.
8. Andreev EM, Shkolnikov VM, Begun A. Algorithm for decomposition of differences between aggregate demographic measures and its application to life expectancies, healthy life expectancies, parity-progression ratios and total fertility rates. *Demographic Research* 2002; **7**:499­522.
9. Andreev EM. Metod komponent v analize prodoljitelnosty zjizni. [The method of components in the analysis of length of life]. *Vestnik Statistiki* 1982; **9**:42-47.
10. Arriaga E. Measuring and explaining the change in life expectancies. *Demography* 1982; **21**:83-96.
11. Pressat R. Contribution des écarts de mortalité par âge à la différance des vies moyennes. *Population* 1985; **4-5**:766-70.

**Table 1 – Countries included in the analysis (N=31) and their data availability**

| **Country** | **Start year** | **End year** | **Additional data sources** |
| --- | --- | --- | --- |
| Austria | 1955 | 2014 |  |
| Belarus | 1981 | 2014 |  |
| Belgium | 1954 | 2015 |  |
| Bulgaria | 1964 | 2010 |  |
| Czech Republic | 1953 | 2016 | For 1953-1985, lung cancer mortality data for the Czech Republic were estimated using data from WHOSIS on former Czechoslovakia. |
| Croatia | 2002 | 2016 |  |
| Denmark | 1951 | 2015 |  |
| Estonia | 1981 | 2014 |  |
| Finland | 1952 | 2015 |  |
| France | 1950 | 2014 |  |
| Germany | 1970 | 2015 |  |
| Germany, East | 1970 | 2014 | For 1970-1972, lung cancer deaths were obtained from the Archive DahlWitz Hoppegarten.  For 1991-2014, lung cancer mortality data from [www.gbe-bund](http://www.gbe-bund) were used. |
| Germany, West | 1956 | 2014 | For 1991-2014, lung cancer mortality data from [www.gbe-bund](http://www.gbe-bund) were used. |
| Greece | 1981 | 2013 |  |
| Hungary | 1955 | 2014 |  |
| Iceland | 1951 | 2016 |  |
| Ireland | 1950 | 2014 |  |
| Italy | 1951 | 2014 |  |
| Latvia | 1980 | 2014 |  |
| Lithuania | 1981 | 2014 |  |
| Luxembourg | 1967 | 2014 |  |
| Netherlands | 1950 | 2016 |  |
| Norway | 1951 | 2014 |  |
| Poland | 1959 | 2014 |  |
| Portugal | 1955 | 2014 | Eurostat data were used to obtain lung cancer deaths for 2004-2006. |
| Russia | 1980 | 2013 | WHOSIS exposure data instead of HMD exposure data were used to calculate lung cancer mortality rates. |
| Slovakia | 1953 | 2014 | For 1953-1991, data for Slovakia were estimated using data from WHOSIS on former Czechoslovakia. |
| Slovenia | 1985 | 2014 |  |
| Spain | 1951 | 2014 |  |
| Sweden | 1951 | 2016 |  |
| Switzerland | 1951 | 2015 |  |
| Ukraine | 1981 | 2012 |  |
| United Kingdom | 1950 | 2015 | Eurostat data were used to obtain lung cancer deaths for 2004-2006. |
