# Supplementary material for: Changing contribution of smoking to the sex differences in life expectancy in Europe, 1950–2014
Source: Eur J Epidemiol. 2020 Jan 22;35(9):835–41. doi: 10.1007/s10654-020-00602-x (PMC7524860; doi:10.1007/s10654-020-00602-x)

**Supplementary Table and Figures**

**Table S1. The absolute and relative contribution of smoking-attributable mortality to the sex difference in life expectancy at birth (e0), 31 European countries, 2014***

| **Country** | **Region** | **Year** | **Sex difference e0**  **(years)** | **Absolute contribution smoking-attributable mortality**  **(years)** | **Absolute contribution nonsmoking attributable mortality**  **(years)** | **Relative contribution smoking-attributable mortality**  **(%)** |
| --- | --- | --- | --- | --- | --- | --- |
| **Denmark** | Northern Europe | 2014 | 4.10 | 0.46 | 3.65 | 11.14 |
| **Finland** | Northern Europe | 2014 | 5.73 | 1.10 | 4.62 | 19.25 |
| **Iceland** | Northern Europe | 2014 | 2.96 | -0.06 | 3.02 | -2.06 |
| **Norway** | Northern Europe | 2014 | 4.07 | 0.41 | 3.66 | 9.96 |
| **Sweden** | Northern Europe | 2014 | 3.70 | 0.02 | 3.68 | 0.52 |
| **Austria** | Western Europe | 2014 | 4.82 | 1.34 | 3.48 | 27.74 |
| **Belgium** | Western Europe | 2014 | 4.95 | 1.83 | 3.13 | 36.86 |
| **Germany** | Western Europe | 2014 | 4.92 | 1.50 | 3.42 | 30.55 |
| **France** | Western Europe | 2014 | 6.16 | 2.26 | 3.91 | 36.62 |
| **Ireland** | Western Europe | 2014 | 4.08 | 0.43 | 3.65 | 10.51 |
| **Luxembourg** | Western Europe | 2014 | 5.36 | 1.67 | 3.68 | 31.25 |
| **Netherlands** | Western Europe | 2014 | 3.42 | 0.79 | 2.63 | 22.96 |
| **Switzerland** | Western Europe | 2014 | 4.18 | 0.85 | 3.33 | 20.44 |
| **United Kingdom** | Western Europe | 2014 | 3.74 | 0.49 | 3.25 | 13.06 |
| **Germany, West** | Western Europe | 2014 | 4.67 | 1.36 | 3.31 | 29.14 |
| **Greece** | Southern Europe | 2013 | 5.24 | 2.86 | 2.39 | 54.45 |
| **Italy** | Southern Europe | 2014 | 4.62 | 1.54 | 3.08 | 33.30 |
| **Portugal** | Southern Europe | 2014 | 6.22 | 2.35 | 3.87 | 37.73 |
| **Spain** | Southern Europe | 2014 | 5.54 | 2.34 | 3.21 | 42.13 |
| **Bulgaria** | Central & Eastern Europe | 2010 | 6.95 | 4.04 | 2.90 | 58.20 |
| **Czech Republic** | Central & Eastern Europe | 2014 | 6.02 | 2.14 | 3.87 | 35.60 |
| **Hungary** | Central & Eastern Europe | 2014 | 6.99 | 3.70 | 3.28 | 53.00 |
| **Poland** | Central & Eastern Europe | 2014 | 7.76 | 3.26 | 4.50 | 42.03 |
| **Slovakia** | Central & Eastern Europe | 2014 | 7.09 | 3.01 | 4.08 | 42.50 |
| **Slovenia** | Central & Eastern Europe | 2014 | 5.72 | 1.96 | 3.75 | 34.33 |
| **Croatia** | Central & Eastern Europe | 2014 | 6.25 | 3.13 | 3.12 | 50.05 |
| **Germany, East** | Central & Eastern Europe | 2014 | 5.99 | 2.18 | 3.81 | 36.45 |
| **Belarus** | Former USSR | 2014 | 10.62 | 4.93 | 5.70 | 46.37 |
| **Estonia** | Former USSR | 2014 | 9.21 | 3.58 | 5.63 | 38.87 |
| **Latvia** | Former USSR | 2014 | 10.17 | 4.61 | 5.56 | 45.35 |
| **Lithuania** | Former USSR | 2014 | 10.72 | 4.88 | 5.84 | 45.54 |
| **Russia** | Former USSR | 2013 | 11.19 | 5.25 | 5.94 | 46.96 |
| **Ukraine** | Former USSR | 2012 | 9.92 | 4.94 | 4.98 | 49.79 |
|  |  |  |  |  |  |  |
| **Weighted average** | Europe | 2014* | 6.97 | 3.03 | 3.94 | 43.52 |

* If the values for 2014 were not yet available, the values for the latest year available were used.

**Figure S1 Changes over time in the absolute contribution* of smoking-attributable mortality to the sex difference in life expectancy at birth (e0), by country, 1950-2014****

**
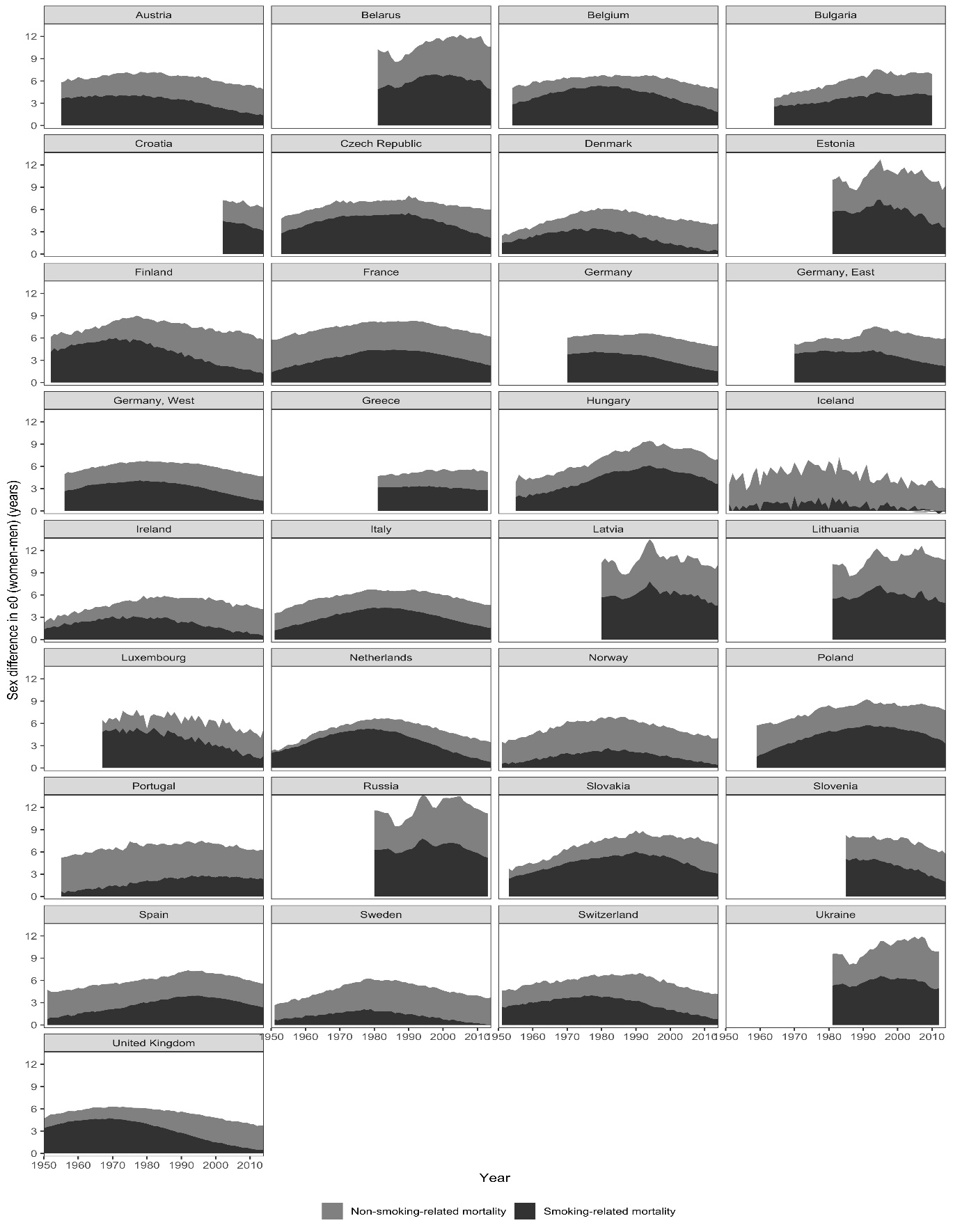
**

* Note that the fluctuations in the absolute contribution of smoking to the sex difference in e0 can be the result of both fluctuations in the smoking-attributable mortality fractions and fluctuations in all-cause mortality. To better distinguish between these effects, please see Figure S2 with the relative contribution of smoking.

** or a shorter period, depending on the data availability

**Figure S2 Changes over time in the relative contribution of smoking-attributable mortality to the sex difference in life expectancy at birth (e0), by country, 1950-2014***


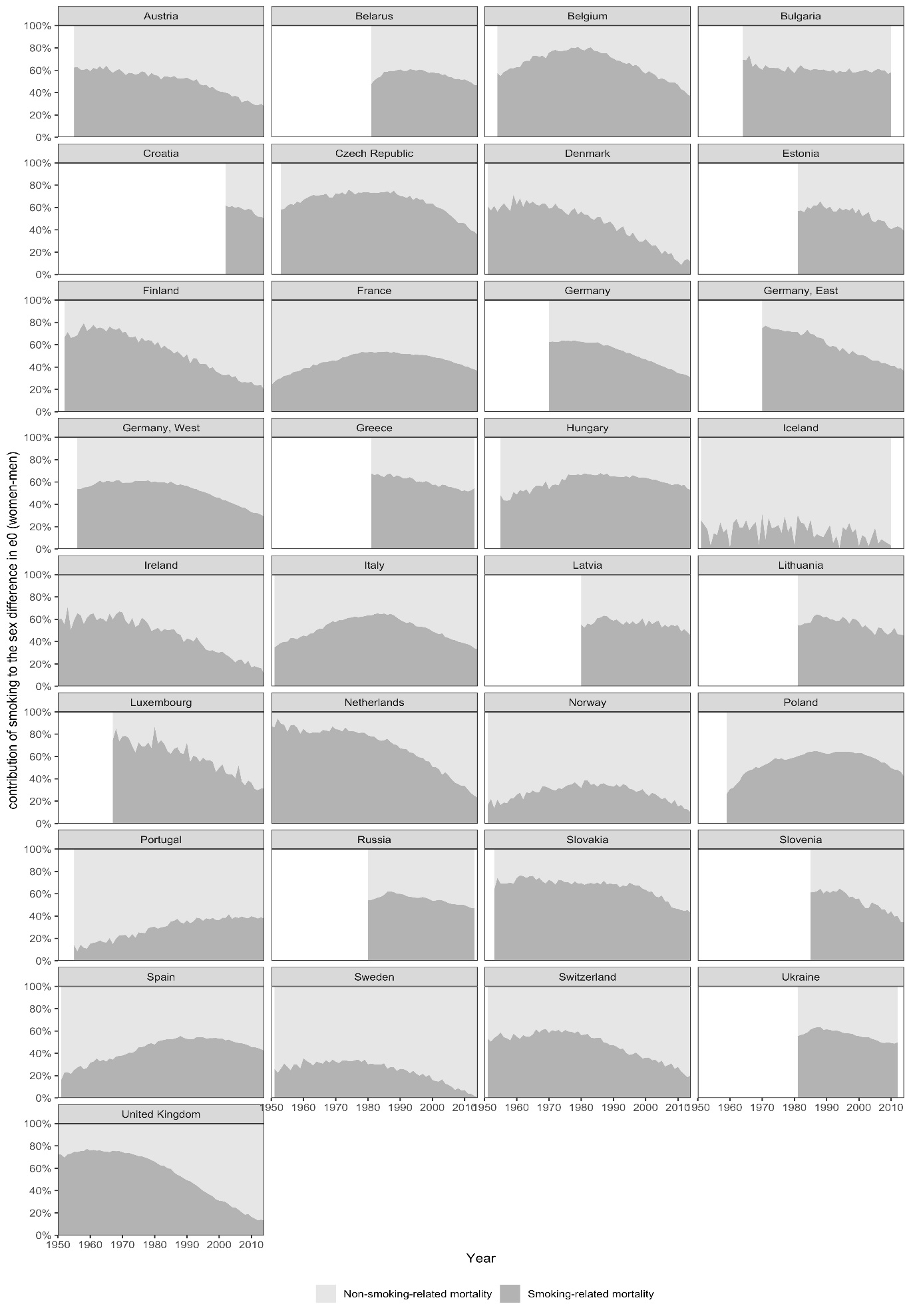

Supplement: Supplementary file 2 — Supplementary file2 (DOCX 678 kb) [file 10654_2020_602_MOESM2_ESM.docx]
